# A step-by-step method to classify corporate sustainability practices based on the Signaling Theory

**DOI:** 10.1016/j.mex.2021.101538

**Published:** 2021-10-03

**Authors:** Norbey Amaya, Mónica López-Santamaría, Yonni Angel Cuero Acosta, Merlin Patricia Grueso Hinestroza

**Affiliations:** School of Management and Business, Universidad del Rosario, Colombia

**Keywords:** Corporate sustainability, Qualitative research, Qualitative content analysis, Intent signals, Necessity signals, Camouflage signals

## Abstract

The “Signaling Theory” is a theoretical framework that investigates the content reported in sustainability reports, considering that through signaling, companies can influence stakeholders’ perceptions, create a competitive advantage and positively impact their corporate image. Signals can be classified into three types: camouflage, intent and necessity. By analyzing their sustainability reports, this study presents a step-by-step approach to classifying sustainability practices reported by companies according to the aforementioned types of signals. We propose a step-by-step approach based on a thematic and qualitative analysis that encourages replication by the research community. Details in the study will validate the proposed method and consider the lessons learned.

• A method is proposed that allows the sustainability practices reported by companies to be classified into camouflage, intent and necessity signals through their sustainability reports.
• A seven-step process for thematic analysis is described based on a qualitative research approach for achieving the above-mentioned goal.
• The classification of sustainability practices into the camouflage typology is a challenging process, as it tends to be biased, hence the identification of this type of signals in comparison with those of intent and necessity requires the adoption of measures that guarantee the reduction of bias on the part of the researcher.

## Graphical abstract

**Figure:**
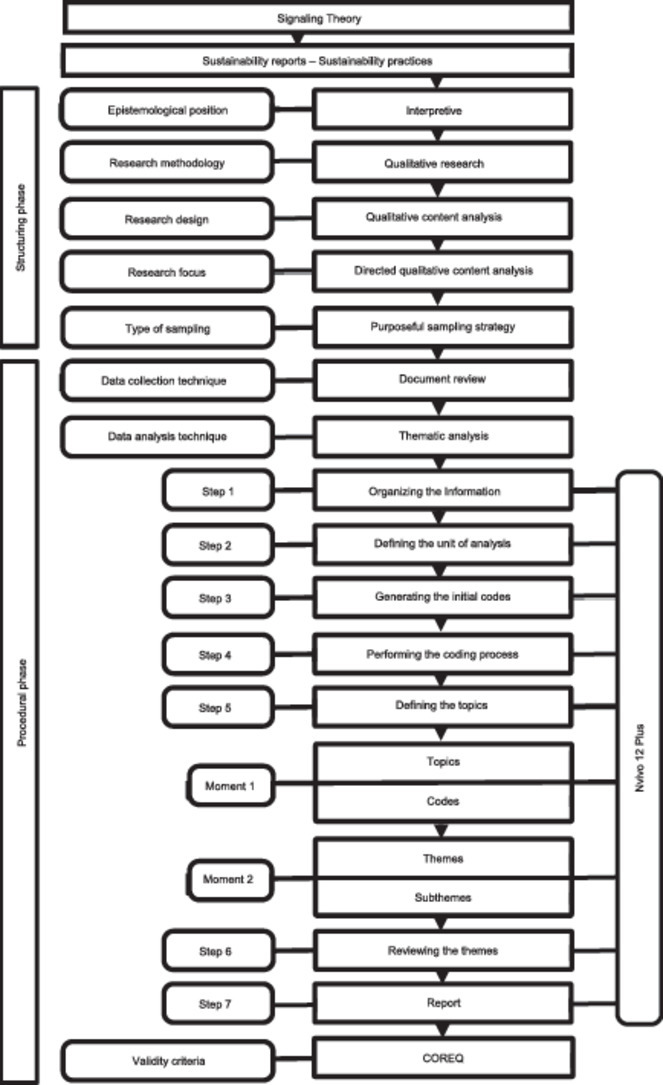

Specifications table

**Table:** 

Subject area	*Economics and finance*
More specific subject area	*Corporate sustainability*
Method name	*Method to classify corporate sustainability practices based on the Signaling Theory*
Name and reference of original method	[4]*Hsieh, H. F., and S.E. Shannon, Three approaches to qualitative content analysis. Qualitative health research, 2005. 15(9): p. 1277-1288.*10.1177/1049732305276687*.*[3]*Braun, V., and V. Clarke, Using thematic analysis in psychology. Qualitative research in psychology, 2006. 3(2): p. 77-101.*10.1191/1478088706qp063oa.
Resource availability	*Software supporting qualitative data analysis: Nvivo 12 Plus*

## Background

The disclosure of sustainability practices has the potential to create value for companies; however, there is no consensus on this approach [6], in fact there are authors who attribute positive effects to disclosure exercises, since they can contribute to organizational transparency by declaring the impact of the companies’ operations. However, detractors argue that sustainability reporting is mainly used as a tool for corporate image management [7] or, worse yet, as a mechanism to draw a veil over the impacts of their actions [8].

The Signaling Theory has been employed [9] to understand the reporting dynamics that companies use and evaluate them in an objective manner. References in current literature reveal the study of sustainability based on the Signaling Theory [6]. Although there is empirical evidence on this latter aspect, some authors have recommended expanding and extending its use to study the disclosure exercise in corporate sustainability reports [10] because signaling helps companies to influence the stakeholders’ perceptions, create a competitive advantage and positively impact their corporate image [11]. Companies can also camouflage information and prevent the acknowledgment of the real evolution of the organizations’ sustainability initiatives [8].

This study presents a step-by-step approach to classifying corporate sustainability practices as set forth in their sustainability reports into three types of signals proposed by Connelly et al. [5] from a qualitative perspective.

To justify the importance of approaching the study of signals in sustainability reports using a qualitative perspective, a search was conducted of the articles published in the Institute for Scientific information web of science (WOS) and Scopus citation indexes, using the terms “sustainability reports” and “Signaling Theory” under “title,” “abstract” and “keywords.” We chose the articles in the aforementioned indexes considering that the former is a citation index frequently used in this type of literature review exercises and that the latter complements the process, since it contains indexed journals that are not found in WOS. This procedure therefore formed an important base of peer-reviewed journals that guaranteed quality information.

The search found eleven research studies, of which only one uses the qualitative content analysis technique to analyze sustainability reports. There is a lack of qualitative analysis in this area of knowledge, despite the fact that this type of analysis of sustainability reports is considered to favor not only the study of the express content disclosed through these documents but also the exploration of the latent content and the undercurrents that may emerge from them [12].

Moreover, although it has been shown that sustainability practices have been studied based on the Signaling Theory, the articles analyzed do not use any type of signal classification. This research reveals the need to classify the signals in order to obtain an improved analysis. Consequently, this study develops a detailed method that incorporates the typology proposed by Connelly et al. [5].

## Phases of the step-by-step construction

### Structuring phase

#### Epistemological position

The epistemological position of this study is interpretivism, a paradigm that is oriented toward the interpretation and understanding of how the social world is constituted [13]. From this position, the researcher thus focuses on comprehensively interpreting all the nuances of a situation [13]. The interpretive tradition is directly related to qualitative research in terms of the approach to the situations studied, the focus on the exploration of social meanings and the interpretation of the texts that bring them together [14].

#### Research methodology

The study of sustainability reports is based primarily on the quantitative description of their content and rarely resorts to a more in-depth analysis of what they communicate and mean [15]. It sought to delve deeper into the content of the reports, using a qualitative approach that consists of varied interpretive practices that make the world visible and simultaneously cause an impact on it [16]. Researchers who apply the qualitative method seek to study phenomena in as natural way as possible so as to substantiate the meanings that people give to them and thus understand them better [16].

#### Research design and approach

Content analysis, which can be both quantitative and qualitative, was employed to develop the step-by-step approach. The quantitative analysis seeks to systematize the frequency of codes according to the content analyzed, while the qualitative analysis is oriented towards uncovering the latent content of a discourse [17]. This study was developed based on a qualitative analysis of content that identifies sustainability practices on the basis of environmental, social and economic dimensions, subsequently classifying them according to the three types of signals proposed by Connelly et al. [5].

Specifically, qualitative content analysis is considered “*a research method for the interpretation of textual data content through the systematic process of classification, coding and identification of themes or patterns*” [4] (p. 1278). This type of analysis is particularly suitable for analyzing documentary information because it constitutes a means to not only analyze the express content of the data but also the latent content, which holds the deep meanings in the texts analyzed [12].

Qualitative content analysis can be understood based on three approaches: conventional, directed and summative. In the first approach, codes emerge during data analysis; in the second approach, the initial codes derive from a theory or previous research results and these codes are defined before and during data analysis. In the third approach, keywords are defined before and during data analysis based on the researchers’ interest, literature review and recurrence of information [4]. This study applied a directed content analysis, since the categorization of sustainability practices was based on the formulation of previous codes based on the Global reporting Initiative (**“GRI”**) indicators [2]. The GRI framework was used because it is recognized as the most referenced system for corporate sustainability disclosure practices [6]. Furthermore, the types of signals are previously established as codes according to the theoretical proposal of Connelly et al. [5].

#### Type of sampling

This study employed a purposeful sampling strategy for the collection of data, which involved the selection of companies or individuals that meet the criteria previously determined by the researchers [18].

### Procedural phase

#### Data collection techniques

A documentary review involves a systematical review or evaluation of both printed and electronic writings [19]. This technique was used in the study through an analysis of secondary sources underlying the sustainability reports issued by the companies analyzed and hosted on their respective websites.

#### Data analysis technique

Based on a previous study [3], the study used thematic analysis as an information analysis technique, which allowed for “*identifying, analyzing* and *reporting patterns (themes) within the data*” [3] (p. 79). This aspect was combined with the implementation of a descriptive coding method, which consists of summarizing in a word or short phrase the basic theme of a piece of text [17].

##### Description of the encoding procedure

The steps of the coding procedure were based on Braun and Clarke's study [3]:

• Step 1 consisted of collecting, preparing and becoming familiar with the information to be coded (determining the number of pages per analyzed report to support this aspect of the research report).
• Step 2 was to specify the unit of analysis. This step is not contemplated in the proposal made by Braun and Clarke [3], however, it is considered important to mention this process to provide more procedural elements to interested researchers. The unit of analysis is “*an expression that encompasses a general meaning*” [20] (p. 89) and refers to the definition of the basic unit of the text to be coded [3]. In this case, the coding started with the interpretation of the sentence or the set of sentences forming a code.
• Step 3 consisted of generating the initial codes to define a dictionary of codes (see Table 1), which was based on the list of GRI indicators [2], the definitions of the corporate sustainability dimensions (environmental, social and economic) and the definitions of the types of signals (camouflage, intent and necessity) proposed by Connelly et al. [5].

**Table 1:** Dictionary of codes.

Code	Definition
**Economic performance**	Direct economic value generated and distributed.
	Economic consequences and other risks and opportunities for the organization's activities that derive from climate change.
	Coverage of the organization's obligations derived from its benefits plan.
	Financial aid granted by government entities.
**Market presence**	Relationship between the initial salary broken down by sex and the local minimum wage in places where significant operations are performed.
	Percentage of senior managers from the local community in places where significant operations are performed.
**Indirect economic consequences**	Development and impact of infrastructural investment and types of services.
	Significant indirect economic impacts and their scope.
**Acquisition practices**	Percentage of spending in places with significant operations corresponding to local suppliers.
**Materials**	Materials by weight or volume.
	Percentage of recycled materials used.
**Energy**	Internal energy consumption.
	External energy consumption.
	Energy intensity.
	Reduction of energy consumption.
	Reductions in the energy requirements of products and services.
**Water and effluents**	Total water withdrawal by source.
	Water sources that have been significantly affected by water collection.
	Percentage and total volume of recycled and reused water.
**Biodiversity**	Owned, leased, managed operating facilities that are adjacent, contain or are in protected and unprotected areas of great biodiversity value.
	Description of the most significant impacts on biodiversity of the protected or unprotected areas derived from activities, products and services.
	Protected or restored habitats.
	Number of species included in national conservation lists whose habitats are in the areas affected by operations according to the level of danger of extinction of the species.
**Emissions**	Direct emissions of greenhouse gases (Scope 1).
	Indirect emissions of greenhouse gases when generating energy (Scope 2).
	Other indirect greenhouse gas emissions (Scope 3).
	Intensity of greenhouse gas emissions.
	Reduction of greenhouse gas emissions.
	Emissions of ozone-depleting substances.
	NO_x_, SO_x_ and other significant air emissions.
**Effluents and waste**	Total water discharge according to its quality and destination.
	Total weight of waste according to the type and method of treatment.
	Total number and volume of significant spills.
	Weight of transported, imported, exported or treated waste that is considered hazardous, and percentage of waste transported internationally.
	Identification, size, protection status and biodiversity value of water bodies and related habitats significantly affected by discharges and runoff from the organization.
**Products and services**	Degree of mitigation of the environmental impact of products and services.
	Percentage of products sold, and their packaging materials recovered at the end of their useful life by product categories.
**Regulatory compliance**	Monetary value of significant fines and number of nonmonetary sanctions for the breach of environmental legislations and regulations.
**Transport**	Significant environmental impacts of the transport of products and other goods and materials used for the organization's activities, as well as the transport of personnel.
**General**	Breakdown of environmental expenses and investments.
**Supplier environmental assessment**	Percentage of new suppliers that were assessed based on environmental criteria.
	Significant, actual and potential negative environmental impacts on the supply chain and measures in this regard.
**Environmental claim mechanisms**	Number of environmental claims that have been submitted, addressed and resolved through formal complaint mechanisms.
**Job**	Number and rate of hiring and average employee turnover classified on the basis of age group, sex and region.
	Social benefits for full-time employees that are not offered to temporary or part-time employees categorized on the basis of locations with significant activity.
	Return to work and retention rates after maternity or paternity leave, classified on the basis of sex.
**Relations between workers and management**	Minimum notice periods for operational changes and their possible inclusion in collective bargaining agreements.
**Health and safety at work**	Percentage of workers who are represented in joint formal health and safety committees for management and employees established to help control and advise on occupational health and safety programs.
	Type and rate of injuries, occupational diseases, days lost, absenteeism and number of fatalities related to work by region and sex.
	Workers whose profession has a high incidence or risk of disease.
	Health and safety issues covered in formal agreements with trade unions.
**Training and education**	Average annual training hours per employee divided on the basis of sex and job category
	Skills management and continuing education programs that promote the employability of workers and help them manage the goals of their professional careers.
	Percentage of employees receiving regular performance and professional development evaluations classified according to their sex and professional category.
**Diversity and equal opportunities**	Composition of the governing bodies and breakdown of the workforce by professional category and sex, age, minority membership and other diversity indicators.
**Equal pay between women and men**	Relationship between the base salary of men and that of women, divided on the basis of professional category and locations of significant activity.
**Evaluation of labor practices of suppliers**	Percentage of new suppliers that were examined based on criteria related to labor practices.
	Significant actual and potential negative impacts on labor practices in the supply chain and actions taken.
**Complaint mechanisms on labor practices**	Number of complaints about labor practices that have been submitted, addressed and resolved through formal complaint mechanisms.
**Investment**	Number and percentage of significant investment contracts and agreements that include human rights clauses or that have been subjected to human rights analysis.
	Employee training hours on policies and procedures related to the aspects of human rights relevant to their activities, including the percentage of trained employees.
**Non-discrimination**	Number of cases of discrimination and corrective actions taken.
**Freedom of association and collective bargaining**	Identification of significant centers and providers in which freedom of association and the right to collective bargaining agreements may be infringed or threatened and measures taken to defend these rights.
**Child labor**	Identification of centers and providers with a significant risk of cases of child exploitation and measures taken to contribute to the abolition of child exploitation.
**Forced labor**	Centers and providers with a significant risk of being the origin of episodes of forced labor and measures taken to contribute to the elimination of all forms of forced labor.
**Security measures**	Percentage of security personnel who have received training in the organization's human rights policies or procedures relevant to operations.
**Rights of the indigenous population**	Number of cases of violation of the rights of indigenous peoples and measures taken.
**Assessment**	Number and percentage of centers that have undergone examinations or evaluations of human rights impacts.
**Assessment of suppliers in the field of human rights**	Percentage of new suppliers that were examined based on criteria related to human rights.
	Significant negative impacts on human rights - real and potential - in the supply chain and measures taken.
**Grievance mechanisms for human rights**	Number of human rights claims that have been submitted, addressed and resolved through formal grievance mechanisms.
**Local communities**	Percentage of centers where development programs, impact assessments and local community participation have been implemented.
	Operations centers with significant negative effects, possible or real, on local communities.
**Fight against corruption**	Number and percentage of centers in which the risks related to corruption and significant risks detected have been evaluated.
	Communication related to and training policies and procedures on the fight against corruption.
	Confirmed cases of corruption and measures taken.
**Public policies**	Value of political contributions by country and recipient.
**Unfair competition practices**	Number of lawsuits for unfair competition, monopolistic practices, or against free competition and their results.
**Regulatory compliance**	Monetary value of significant fines and number of nonmonetary penalties for the breach of legislations and regulations.
**Evaluation of the social impact of suppliers**	Percentage of new suppliers that were examined based on criteria related to social impact.
	Significant and potential negative impacts on the society in the supply chain and measures taken.
**Social impact grievance mechanisms**	Number of complaints about social impacts that have been submitted, addressed and resolved through formal complaint mechanisms.
**Health and customer safety**	Percentage of categories of significant products and services whose health and safety impacts have been evaluated to encourage improvements.
	Number of incidents derived from non-compliance with regulations or voluntary codes related to the impacts of products and services on health and safety during their life cycle, broken down according to the type of result of said incidents.
**Labeling of products and services**	Type of information required by the organization's procedures related to the information and labeling of its products and services and percentage of categories of significant products and services that are subject to such requirements.
	Number of breaches of regulatory and voluntary codes related to information and labeling of products and services, broken down according to the type of result.
	Survey results to measure customer satisfaction.
**Marketing communications**	Sale of prohibited or disputed products.
	Number of cases of non-compliance with the regulations or voluntary codes related to marketing communications, such as advertising, promotion and sponsorship, broken down according to the type of result.
**Customer privacy**	Number of substantiated claims regarding privacy breach and customer data leakage.
**Regulatory compliance**	Cost of significant fines for non-compliance with regulations and legislations related to the supply and use of products and services.
**Environmental dimension**	Impacts of a company on living and non-living natural systems, such as materials, energy, water, biodiversity, emissions, effluents and waste, products and services, or regulatory compliance [2].
**Social dimension**	The effects that a company's activities have on the social environment encompasses four subcategories: labor practices and decent work, human rights, society and product responsibility [2].
**Economic dimension**	Impacts of the organization on the economic situation of stakeholders and economic systems at local, national and international levels, characterized by a focus on the company's financial situation, including issues such as economic performance, market presence, indirect economic consequences, or procurement practices [2].
**Camouflage signals**	These are conceived as those that hide a possible liability of the organization and seek to divert attention from a potential vulnerability that could lead to corporate action [5].
**Intent signals**	Future actions that are referred to in the means of organizational disclosure and may be conditional on the recipient's response [5].
**Necessity signals**	These are intended to communicate the requirements with which the company must comply [5].

Source: Prepared by the authors based on GRI [2] and Connelly et al.’s study [5].
• Step 4 was related to the coding of the information provided by the sustainability reports. The information was thus classified according to the previously established codes, and it was conceived as a first-order coding performed independently by the researchers.
• Step 5 consisted of specifying the themes, defined as “*an abstract entity that provides meaning and identity to a recurring reference and its manifestations*” [21] (p. 362). This step was configured in two moments to determine the themes in which the codes were grouped and hierarchize the coded information. In Moment 1, the codes defined based on the GRI indicators were grouped into each of the corporate sustainability dimensions (environmental, social and economic); at this stage, these dimensions were grouped into themes. In Moment 2, the previously coded sustainability practices were grouped into signals (camouflage, intent and necessity). Thus, in Moment 2, the GRI indicators have the status of codes, the sustainability dimensions play the role of subthemes and the signals act as themes. This moment was conceived as second-order coding, which sought the most significant or frequent initial codes to identify emerging categories and group similar codes together to synthesize the number of categories [17]. This process was performed independently by the researchers.
• Step 6 was to review the themes into which the codes had been grouped to ensure that they had been well classified. This aspect involved meetings of the researchers and consultation among them based on the literature review.
• Step 7 consisted of the preparation of the research report wherein fragments of the coded information were referenced to support the results achieved.
• Specialized software for qualitative information (Nvivo Plus 12) was used for the information analysis, as it is considered a tool that enables information management, its coding and text retrieval.

### Validity criteria

Validity criteria were based upon some of the requirements suggested by the consolidated criteria for reporting qualitative research (COREQ) [1], which are described in Table 2.

**Table 2:** Validity criteria.

Criteria considered in COREQ	Fulfillment of the criteria
How many data coders coded the data?	The coding process was conducted by two researchers, which involved double coding and confirmation of the code and theme understanding through an agreement between them.
Did authors provide a description of the coding tree?	The coding tree was described using the code dictionary (see Table 1) and the category map; see https://padlet.com/sustainabilityresearch036/pscirgjkt7 × 6c6so.
Were themes identified in advance or derived from the data?	Although the codes were previously defined, responding to a deductive creation of codes [22], this did not mean rejecting the codes that emerged from the recurrence of the information.
What software, if applicable, was used to manage the data?	The Nvivo 12 Plus qualitative data analysis software was used.
Were the data and the findings consistent?	Consistency can be assessed by reviewing the research article entitled “*Sustainability disclosure practices as seen through the lens of the Signaling Theory: A study of companies listed on the Colombian Stock Exchange*”
Were major themes clearly presented in the findings?	Clarity can be assessed by reviewing the research article entitled “*Sustainability disclosure practices as seen through the lens of the Signaling Theory: A study of companies listed on the Colombian stock exchange*”.

Source: Based on Tong, et al. study [1] (p. 352).

## Step-by-step application based on a research exercise

This method was applied to classify and analyze the sustainability practices of companies listed on the Colombian stock exchange (*Bolsa de Valores de Colombia* - **“BVC”**) through the typology of signals (camouflage, intent and necessity) proposed by Connelly et al. [5].

We chose the organizations that participated in this study using purposeful sampling strategy criteria based on the following determinants: (a) organizations listed on the BVC, as these companies must issue sustainability reports [23], (b) companies with market capitalization of over fifty million Colombian pesos (COP 50,000,000) (approximately USD 14,000) because they are considered to be part of the top reference group and (c) the possibility of virtual access to the company's reports. Thus, of the 176 companies registered with the BVC, 43 met the guidelines established by the researchers.

Table 3 below describes the research conducted based on the seven steps described in the section entitled “*Data analysis technique*” of the procedural phase.

**Table 3:** Description and examples of the steps taken to classify corporate sustainability practices, based on the Signaling Theory.

Steps	Procedure	Example
1	A database was organized with the data of the companies analyzed, considering the identification number of the organization, the corporate name, its economic sector, its age, its region, the type of report issued, the number of pages of the report and a brief description of the company. The thematic analysis involved the review of 43 corporate sustainability reports, a total of 7527 pages.	To see the database developed, visit the following link: https://padlet.com/sustainabilityresearch036/pscirgjkt7 × 6c6so
2	The sentence or group of sentences was defined as a unit of analysis.	“*Due to ethnic relations, we have no complaints before the human rights offices of Colombia, Brazil, Guatemala and Peru*” (Organization Number 35).
3	A code dictionary was developed based on the GRI indicators, sustainability dimensions and the definitions of the types of signals proposed by Connelly et al. [5].	See Table 1.
4	The coding process enabled the emergence of new codes. In this study, due to the recurrence of information, a new code was configured and named “Workers’ welfare.” In the example, the conceptual definition that was agreed among the researchers for this emerging code is mentioned. It is important to remember that this step was performed individually by the researchers (first-order coding) and was refined in the meeting considered during Step 5.	Stability compatible with economic dynamism, safety, health at work, assessment of individual careers and the development of inherent competences, opportunities to acquire and maintain professional qualifications, guarantee of adequate income, protection of collective representation and labor market security (Boyer, 2002 as cited in [24].
5	The themes into which the codes were grouped were defined, always taking as a reference the theoretical framework on which the research was based. This process was structured in two stages. In Stage 1, the defined codes were organized (based on the GRI indicators) in each of the corporate sustainability dimensions (environmental, social and economic). In Stage 2, the previously coded sustainability practices were grouped into types of signals (camouflage, intent and necessity). Thus, the GRI indicators have the status of codes, the sustainability dimensions play the role of subthemes and the signals act as themes. This process (second-order coding) was conducted independently by the researchers.	**Stage 1Theme:** Environmental dimension**Code:** Biodiversity**Reference**:“*In Colombia, we have been working with three local universities—Cauca, Valle and Quindío—since 2009, studying flora and fauna populations in and around our protected forests and forest plantations*” (Organization Number 15).**Stage 2Theme:** Intent signal**Subtheme:** Environmental dimension**Code:** Biodiversity**Reference:**For the year 2019, the Company will analyze different projects to boost hydroelectric plants or other renewable energy sources and reduce diesel consumption through own generation (Organization Number 40).
6	The themes into which the codes were grouped (second-order coding) were revised based on the consensus among the researchers.Due to the recurrence of information, a new theme was configured and named “Sign of prominence,” and the emerging category “Workers’ Welfare” was associated with the theme “Social Dimension.” The conceptual definition that was agreed among the researchers for the emerging theme is recorded in the right-hand column of the table under “Example,” and the category map that was created after the meeting between the researchers is shared.	Degree of company's acknowledgment in its organizational field based on its position in rankings, certifications achieved and affiliation with high status actors [25].To see the category map, visit the following link:https://padlet.com/sustainabilityresearch036/pscirgjkt7 × 6c6so
7	A research article was written based on the research report.	Research paper title: “*Sustainability disclosure practices as seen through the lens of the Signaling Theory: A study of companies listed on the Colombian Stock Exchange*”.The research results showed that the majority of signals identified were those of intent, followed by those of necessity and finally those of camouflage.The camouflage, intent and necessity signals refer mostly to sustainability practices pertaining to the social dimension.Moreover, the study proposes that camouflage signals can be classified into two types: 1) those that seek to hide some type of corporate social responsibility or divert attention from potential harm that may be caused by the company and 2) those that seek to highlight organizational attributes and achievements.This finding shows that sustainability reporting not only aims to reduce information asymmetry between companies and stakeholders but also to impact organizational reputation by promoting organizational attributes and achievements.

Source: Based on the studies of Connelly et al. [5] and Rindova et al. [25].

## Lessons learned

The lessons learned from this step-by-step process are described below. An initial sample of the organizations to be analyzed was used to exemplify the step-by-step process, however, not all the sustainability reports of this sample could be accessed, as some were not available in digital format and others could not be read by the qualitative data analysis software used. These conditions should therefore be checked beforehand to determine a sample that is accessible.

Furthermore, the sustainability reports found in this study have different names, therefore it is advisable to group them broadly in a generic category that includes labels such as annual, management, integrated, shared value, social balance, social value and integrated management reports [7].

One of the main challenges faced by researchers in the coding process was to identify the information that could be classified as camouflage signals, since their definition favors interpretation biases. Thus, Table 4 provides comments which are hoped will serve as clues to researchers who may face this same challenge and also shows examples of references that support this type of signal.

**Table 4:** Examples and comments regarding references supporting Camouflage signals.

Theme	Sub-theme	Code	Reference example	Comments
**Camouflage signals:** Conceived as being those that hide a possible liability of the organization and seek to divert attention from a potential vulnerability that could lead to some corporate action [5].	**Environmental dimension**	**Biodiversity**	“*During the company's operations in 2018, there were no events of water discharges or runoffs that could significantly affect biodiversity*.”(Organization Number 13).	The first type of Camouflage signals identified are those sentences that used expressions such as those highlighted and in italics, i.e., the action being disclosed is not considered serious, even if it has somehow caused some damage or involved blame.
		**Regulatory compliance**	“*During 2018 the Company had no significant sanctions or fines associated with the non-compliance of environmental laws or regulations.*” (Organization Number 23).	
	**Social dimension**	**Company**	“*The contribution made in 2018 of COP 129,562,941 was used to start the construction of the Intensive Care Unit and Pediatric Integral Recovery Unit of Fundación Cardioinfantil, an area that has increased its capacity to care for children with highly complex diseases by 75%. More than 720 boys and girls are cared for over a year*.” (Organization Number 4).	Another way of identifying Camouflage signals agreed upon by the researchers was related to the practices reported by the companies that are not associated with the core business and are presented as corporate social responsibility actions; for example, the company cited provides air transport services in Colombia. To strengthen the identification of this type of signal, we recommend having sufficient information on the core operations of each organization (refer to the development of the database of the companies analyzed mentioned in Step 1).
	**Economic dimension**	**Indirect economic consequences**	“*Due to the timely payment of taxes and obligations, we are one of the companies that contribute the most to regional development*.” (Organization number 3).	Another way to identify camouflage signals agreed among the researchers had to do with those references of law enforcement actions performed by the companies and presented as a good practice.

Source: Based on Connelly et al. [5].

This step-by-step approach should contribute to the development of further research on the dissemination of sustainability practices based on the Signaling Theory.

## Conclusion

Based on the Signaling Theory, this article describes a seven-step procedure that facilitates the classification of business sustainability practices in their social, economic and environmental dimensions. This classification was obtained from an analysis of the sustainability reports of a group of 43 companies that are traded on the Colombian stock exchange (“BVC”), using the thematic analysis technique.

By documenting the steps developed to classify sustainability practices, the article makes relevant contributions to the field. The first contribution is the methodology itself, since it highlights the need to incorporate an additional step to the proposal developed by Braun and Clarke [3]. This complementary step consists of defining the unit of analysis to carry out the thematic analysis, in this case represented in the sentence or in the set of sentences that form a code. The proposed methodology also incorporates examples of the criteria to be employed when carrying out the coding of the Camouflage signals, which in the opinion of the authors of this article are the most problematic to code, because their conceptual definition is limited, unlike Signals of intent and need. This guidance on the coding of camouflage signals is intended to favor the replicability of the research by other researchers since the authors of this work are of the opinion that they may be more subject to interpretation biases. Finally, the methodology incorporates validity criteria that are not commonly applied in research based on thematic analysis. The use of certain COREQ (Consolidated Criteria for reporting qualitative research) criteria is particularly recommended since they give the research a more solid foundation.

## Acknowledgment

The authors would like to thank Universidad del Rosario (https://www.urosario.edu.co) for its financial support in the copyediting service and the technical support of Enago^TM^ in this same process.

## Declaration of Competing Interest

The authors represent that they have no known competing financial interests or personal relationships that could have appeared to influence the work reported in this paper.
